# Structural differences in impaired verbal fluency in essential tremor patients compared to healthy controls

**DOI:** 10.1002/brb3.722

**Published:** 2017-05-15

**Authors:** Esther A. Pelzer, Christian Nelles, David J. Pedrosa, Carsten Eggers, Lothar Burghaus, Corina Melzer, Marc Tittgemeyer, Lars Timmermann

**Affiliations:** ^1^ Max Planck Institute for Metabolism Research Cologne Cologne Germany; ^2^ Department of Neurology University Hospital Cologne Cologne Germany

**Keywords:** corpus callosum, essential tremor, magnetic resonance images, precuneus, tract‐based spatial statistics, verbal fluency, voxel‐based morphometry

## Abstract

**Objective:**

We wanted to identify differences in grey and white matter in essential tremor patients compared to controls in the non‐motor domain, using the example of impaired verbal fluency.

**Background:**

A disturbance of verbal fluency in essential tremor patients compared to healthy controls is behaviorally well described.

**Methods:**

Voxel‐based morphometry and tract‐based spatial statistics were used to analyze structural differences in grey and white matter in 19 essential tremor patients compared to 23 age‐ and gender‐matched controls.

**Results:**

Several significant observations were made. (I) There was less grey matter in the predominantly right precuneus in the essential tremor group compared to controls [*p *< .001]. (II) In ET patients mean, axial, and radial diffusivity values broadly correlated with the tremor rating scale, pronounced in fronto‐parietal regions [*p *< .05]. (III) In ET patients there was a significant decline in fractional anisotropy values in the corpus callosum in the correlation with verbal fluency results [*p *< .05]; by inclusion of the tremor rating scale as covariate of no interest this significance was however diminished to a tendency (*p *< .1). No significant results were found in these within‐group correlations in grey matter analyses for ET patients (*p *> .05).

**Conclusion:**

The present results indicate that non‐motor symptoms such as verbal fluency (VBF) in ET have a structural substrate; their reproduction requires the integration of potential environmental plasticity effects, differentiation into individual clinical subtypes and a careful handling with methodological peculiarities of structural MR imaging.

## 1 INTRODUCTION

Essential tremor (ET) has been primarily characterized by its motor symptoms, which can manifest in heterogeneous severity with a devastating impact on patients' quality of life (Chandran & Pal, 2013). Recently, however, an increasing body of evidence indicates additional non‐motor symptoms in ET patients (Benito‐León & Louis, 2006; Louis, 2010). An impairment of executive functions in general and verbal fluency (VBF) in particular has already been reported (Tröster et al., 2002). However, the underlying mechanisms resulting in these VBF deficits remain elusive.

To elucidate structural differences in ET patients compared to healthy controls, a number of studies have applied voxel‐based morphometry (VBM) for grey matter analyses and tract‐based spatial statistics (TBSS) for white matter analyses. VBM investigates voxel‐wise differences in the local grey matter topography in one population related to one clinical score, or between several populations; TBSS aims to improve the sensitivity, objectivity, and interpretability of analysis of multi‐subject diffusion imaging studies by (1) carefully tuned nonlinear registration and (2) projection onto an alignment‐invariant tract representation. As a result of TBSS analyses fractional anisotropy (FA) is a measurement of the degree of anisotropy in the apparent diffusion (scaled between 0‐1) and is, for example, a non‐ specific biomarker of neuropathology and microstructural architecture. Although radial (RD), axial (AD) and medial diffusivity (MD) are specific for the type of change (e.g., axonal damage or demyelination), the interpretation of these parameters is, however, challenging (for further details please see Alexander, Lee, Lazar, & Field, 2007).

Due to the fact that the underlying pathology of the disease is still only partially understood (Benito‐Leon, 2014), and the affected brain areas are still under debate, these statistical methods provide a good solution to primarily detect disease‐specific abnormalities (Benito‐León & Louis, 2006).

In the analysis of motor symptoms, a grey matter involvement bilaterally in the region of the temporo‐parietal junction and right middle occipital cortex has been found (Daniels et al., 2006); a cerebellar involvement and changes in frontal lobes have also been described by Quattrone et al. (2008). Benito‐León et al. (2009); Lin et al. (2013) additionally reported changes in the right insula, the caudate nucleus, precuneus, and precentral gyrus. Regarding white matter analyses, more studies report large fronto‐parietal changes, changes in the internal and external capsule, left superior cerebellar peduncle, right corticospinal tract, cerebellum, thalamus, and brainstem (Klein et al., 2010; Nicoletti et al., 2010; Shin, Han, Kim, & Lee, 2008). Thus, it may be surmised that all of these different regions may not solely be related to the motor domain as reflected by the tremor network, but may also refer to the non‐motor domain.

As far as we are aware, few studies have investigated structural differences due to non‐motor symptoms in ET compared to controls [e.g., Bhalsing et al. (2015)], and none have specifically focused on VBF. Therefore, this study aimed to explore the specific correlations between performance in VBF task and structural magnetic resonance images (MRI).

## 2 METHODS

The study was approved by the local ethics committee (13‐090) and carried out in accordance with the Declaration of Helsinki. All patients gave written informed consent prior to participating. From the outpatient clinic of our tertiary care hospital, a total number of 24 ET patients and 24 age‐ and gender‐matched controls were recruited for the experiment.

All participants were right handed, with a laterality index >50 on the Edinburgh Handedness Inventory (Oldfield 1971), and were first evaluated for depression using the Beck Depression Inventory [BDI‐II; Beck, Steer, Ball, and Ranieri (1996)]. Participants with moderate‐to‐severe depression, reflected by BDI‐II values >19 points, were excluded from the study. All participants were also tested using cognitive functioning screening, the DemTect [for further details, please see Kalbe et al. (2004)]. The DemTect is short (8–10 min), easy to administer, and its transformed total score (maximum 18) is independent of age and education. The DemTect helps in deciding whether cognitive performance is adequate for age (13–18 points), or whether mild cognitive impairment (9–12 points) or dementia (8 points or below) should be suspected. The assessment of VBF was studied using the DemTect's subtest (“the supermarket”), which explores semantic VBF. The instructions of the supermarket task were the following: The subjects were allotted 60 s to name different items, which can be bought in a supermarket. The verbal responses were recorded. For every named word one cross was set in the test sheet. Iterative words were not included in the assessment. If the patient stopped before time was running out, the investigator could tell the patient to name further items. A time watch determined the time after 60 s. Beside the VBF values, values of word list, number transcoding task, digit span backwards and delayed recall of word as part of the DemTect were acquired.

To assess general psychomotor abilities, participants additionally accomplished the Hallstead finger tapping test five times for each hand in random order, allowing estimates of tapping frequencies (Reitan & Wolfson, 1986). Reaction times, in turn, resulted from a task in which participants pressed a button following a visual cue. Finally, ET patients were videotaped for clinical tremor rating. Tremor severity was analyzed under double‐blind conditions by three experienced raters (D.P., C.E., and L.B.) using the Fahn‐Tolosa‐Marin Tremor Rating Scale [TRS; Fahn, Tolosa, and Marin (1993)]. All included ET patients had an unambiguous diagnosis according to the *Consensus Statement of the Movement Disorder Society on Tremor* (Deuschl, Bain, & Brin, 1998). Consistency in the TRS was assessed using a two‐way mixed, average‐measured intra‐class coefficient correlation [ICC; (McGraw & Wong, 1996)].

Subsequently, MR images were acquired using a 3T Siemens Trio Scanner (Siemens Healthcare, Erlangen, Germany) with a maximum gradient strength of 40 mT/m. In total, three data sets were acquired, two high‐resolution structural and one diffusion‐weighted MRI set. The high‐resolution structural data was obtained using a twelve‐channel array head coil with a whole brain field of view (FoV). Thereby, the former two recordings comprised (1) MDEFT‐3D (T1‐weighted, TR = 1930 ms, TI = 650 ms, TE = 5.8 ms, image dimension = 256 × 256 × 128, sagittal slices with a resolution of 1 × 1 × 1.25 mm³, flip angle 18°) and (2) RARE (T2‐weighted, TR = 3200 ms, TE = 482 ms, image dimension = 240 × 256 × 176, sagittal slices with a slice resolution of 1 × 1 × 1 mm³). The diffusion MRI was acquired using spin‐echo echo planar imaging (SE‐EPI, TR = 11200 ms, TE = 87 ms, FoV 220 × 220 × 153 mm^3^, bandwidth 1628 Hz/pixel, matrix 128 × 128 × 90) with double spin echo preparation and a 32‐channel array head coil. Diffusion weighting was isotropically distributed along 60 gradient directions (b‐value = 1000 s/mm^2^) and was acquired within six blocks. Each of those blocks consisted of an initial volume without any diffusion weighting and 10 diffusion‐weighted images.

The data analyses were performed using FMRIB Software Library tools (Smith et al., 2004): (1) TBSS analysis for white matter, and (2) VBM analysis for the examination of grey matter involvement. Before the application of TBSS‐ and VBM‐analyses, intracranial volumes were measured with SIENAX (Smith et al., 2002).

### 2.1 TBSS analysis

Initially, diffusion‐weighted images were motion corrected by registering each volume onto the very first non‐diffusion weighted image of the measurement by applying a rigid body registration (6 degrees of freedom). To reduce registration and interpolation artifacts, the registration of each single volume was performed in two steps. In a first step, the diffusion‐weighted volumes of each block were registered onto the non‐diffusion weighted image of the block. In the second step, the non‐diffusion weighted volumes were registered onto the very first non‐diffusion weighted image, which therefore serves as the subject‐specific reference space of the whole diffusion measurement. For each volume, the two transformation matrices of these registrations were concatenated and finally applied. Consecutively, the tensor reconstruction was performed using DTIFIT, a component of the FMRIB's Diffusion Toolbox. In this reconstruction process, additional maps of FA, RD, AD, and MD were generated.

After reorienting the obtained FA images to Montreal Neurological Institute (MNI) standard space, they were slightly eroded and the most inferior slices were zeroed for data control. Subsequently, the FA images of all subjects were registered onto the FMRIB58_FA standard‐space image using FMRIB's non‐linear image registration tool [FNIRT; Jenkinson, Beckmann, Behrens, Woolrich, and Smith (2012)] with alignment to 1 mm resolution. The mean FA image was generated and thinned, resulting in a mean FA skeleton that represented the centers of all tracts common to the group. Subsequently, this skeleton was thresholded at an FA value of 0.2 to omit statistical interference by grey matter. Aligned, individual FA data was projected onto this skeleton and the resulting data were passed into voxel‐wise general linear modeling across subject statistics. A nonparametric voxel‐by‐voxel permutation test with 5000 permutations was used to assess group‐related differences using threshold‐free cluster enhancement (FSL‐TFCE). For statistical inference, the results were initially observed at a significance level of *p *< .05, corrected for multiple comparisons across space. In addition MD‐, AD‐ and RD‐maps were compared in an analogous fashion.

For statistical analyses the following designs (according to FSL‐GLM) were set up:

Design I (“Two group difference”): A between‐group test between ET patients and controls was designed with age and gender as covariate/ factor of no interest.
Design II (“Single‐Group Average with Additional Covariate”): We then performed patient specific subgroup analyses where we correlated grey and white matter values with: (a) the TRS to explore the tremor‐related network with age and gender as covariate/ factor of no interest; (b) behaviorally acquired VBF values with age, gender, and handedness included as additional explanatory variables; (c) behaviorally acquired VBF values with age, gender, handedness, and tremor included as additional explanatory variables. For the controls we could only test the within‐group correlation for design IIb, because the controls had a TRS (tremor rating scale) score of 0; this made the design matrix for contrast IIa and IIc inefficient.

### 2.2 VBM analysis

Voxel‐based morphometry analysis implemented in FSL was performed to establish grey matter abnormalities. To that end, individual T1‐weighted images were used as the structural basis for the analyses. In a first step, images were brain extracted using FSL‐BET, segmented by FSL‐FAST, and a study‐specific 4D‐grey matter template was created. Therefore, a nonlinear registration was adopted with transformations of the images to the ICBM MNI template. In a final step, individual grey matter images were nonlinearly registered to the study‐specific template and spatially smoothed with 3 mm kernel; this kernel was recommended due to the voxel size of the MDEFT 3D image (1 × 1 × 1.25 mm^3^). Again, a nonparametric voxel‐by‐voxel test with 5000 permutations was used to assess group‐related differences using TFCE. For the between‐group tests, the same three designs as for the TBSS analyses were applied (see above).

## 3 RESULTS

### 3.1 Clinical and general data

Of those recruited, 19 ET patients and 23 control subjects were included in the MR analyses. Five ET patients and one control subject had to be excluded due to abortion of MRI acquisition [caused by claustrophobia, for example, or due to movement and registration artifacts in MRI], symptoms of depression (BDI >19 points) or dementia (DemTect <9 points).

In the DemTect, the majority of participants had values >12 points, excluding severe dementia; only two patients had a mild cognitive impairment in the DemTect (9‐12 points). In the VBF task “the supermarket” ET patients produced significantly fewer words compared to healthy control subjects (*p *< .001). Additionally we found significant differences between patients and controls in “the word list” (*p *< .05), testing verbal recall. By correlating VBF with results of “ the word list” we found a high dependency between these both measurements (*p *< .001). Tremor severity scores in the ET group ranged from 8 to 75 on the TRS (mean 24.98 ± 2.76 SEM), indicating mild to medium motor symptoms, predominantly with a holding tremor component. The resulting intra‐class correlation (ICC = 91.2; confidence interval [CI]  = 90.0; 92.3) for the TRS ratings among the three raters indicated high concordance. No significant difference was found in the reaction time between ET patients and controls (*p *> .05); the finger tapping (as indicator for motor performance) was bilaterally significantly reduced in the patients group compared to healthy controls (*p *< .05). Clinical data and the results of applied tests are summarized in Table 1.

**Table 1:** General demographics for ET‐patients and the age‐ and sex‐matched control group (all values in mean ± SEM)

	Controls (*n *= 23)	ET‐patients (*n *= 19)	Effect size^a^	*p*‐value^b^
Age (in years)	50.93 ± 3.33	49.47 ± 3.51	−.13	.755
Educational years	15.52 ± 0.42	14.74 ± 0.51	.48	.273^c^
Gender ♀:♂	11:12	10:9	–	–
Reaction time (ms)	225.75 ± 11.38	235.11 ± 12.90	−.22	.589
Tapping Test right hand (per min)	58.34 ± 1.85	48.00 ± 3.77	1.04	**.014**
Tapping Test left hand (per min)	51.41 ± 2.14	43.08 ± 3.49	.85	**.043**
DemTect	17.35 ± 0.29	16.68 ± 0.54	.46	.413^c^
Word list	15.78 ± 0.51	13.32 ± 0.72	1.14	**.006** c ^**,**^ d
Number transcoding task	3.91 ± 0.06	3.89 ± 0.07	.08	.842^c^^,^ d
Verbal fluency	29.13 ± 0.39	25.47 ± 1.02	1.41	**.001** d
Digit span backwards	5.35 ± 0.16	5.68 ± 0.13	−.63	.103^c^^,^ d
Delayed recall of word list	7.65 ± 0.39	6.68 ± 0.51	.62	.135^d^
TRS	–	24.50 ± 3.41	–	–

Significant results with p < .05 are displayed in bold.

a Effect sizes according to Cohen's *d*.

b If not indicated specifically, group differences were computed using an independent samples *t*‐tests as parameters showed normal distribution in a Kolmogorov‐Smirnov tests. Level of significance was α = 0.05.

c Due to non‐parametric distribution, group differences were tested using a Wilcoxon‐Mann‐Whitney test.

d For the control of family‐wise error for DemTect subtests, Holm‐Bonferroni correction was applied.

### 3.2 Imaging data

In the SIENAX analysis, intracranial brain volumes (based on T1‐weighted images) did not significantly differ between the two groups (*p *> .05).

#### 3.2.1 Grey and white matter differences in the group comparison

**Figure 1:**
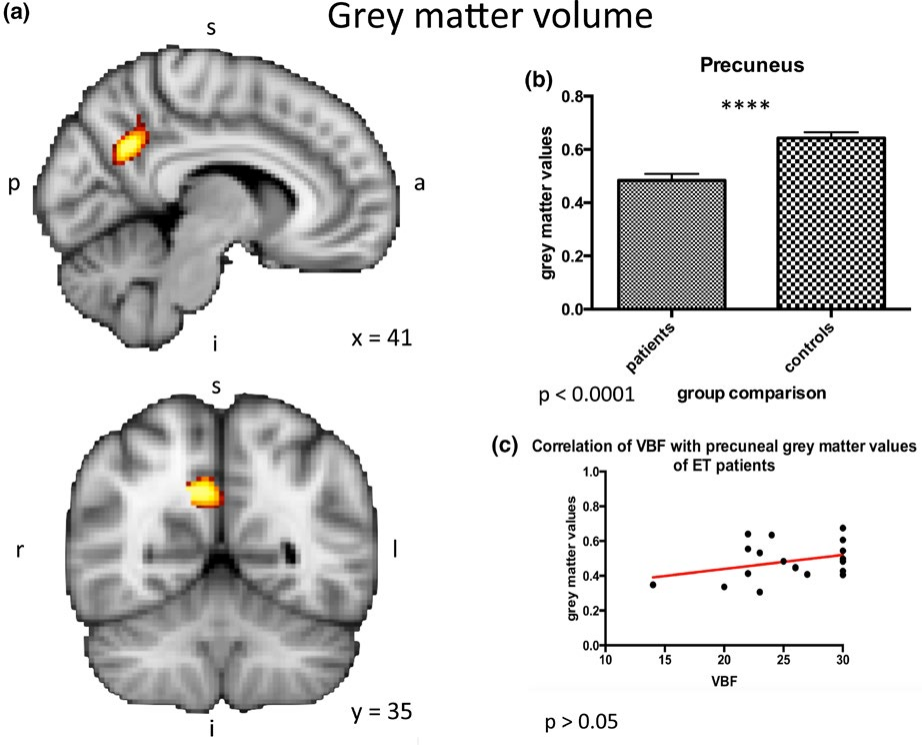
Grey matter volume. (a, b) Group differences between ET patients and age‐matched controls. Design I: Grey matter analysis in the group comparison of ET‐patients and healthy controls revealed high significant differences in the right precuneus (*p *< .001). (c) Correlation of VBF with precuneal grey matter values of ET patients. In the right precuneus, where the group difference in grey matter volume between patients and controls was greatest, greater grey matter volume tended to be associated with greater VBF scores on average in the patients' group (although, of course, non‐significantly 1 C; *p *> .05). ET, essential tremor

Two‐group testing (design I) revealed no significant white‐matter differences (*p *> .05). In contrast, TFCE‐corrected VBM analyses (design I) without age and gender as covariate factor of interest revealed a decrease of grey matter in the bilateral precuneus (right > left) of ET patients compared to controls (*p *< .001, cf. Figure 1a,b). In this region greater grey matter volume tended to be associated with greater VBF scores on average in the patients' group (although, of course, non‐significantly; Figure 1c; *p *> .05).

#### 3.2.2 Grey and white matter differences in ET patients regarding the tremor network

**Figure 2:**
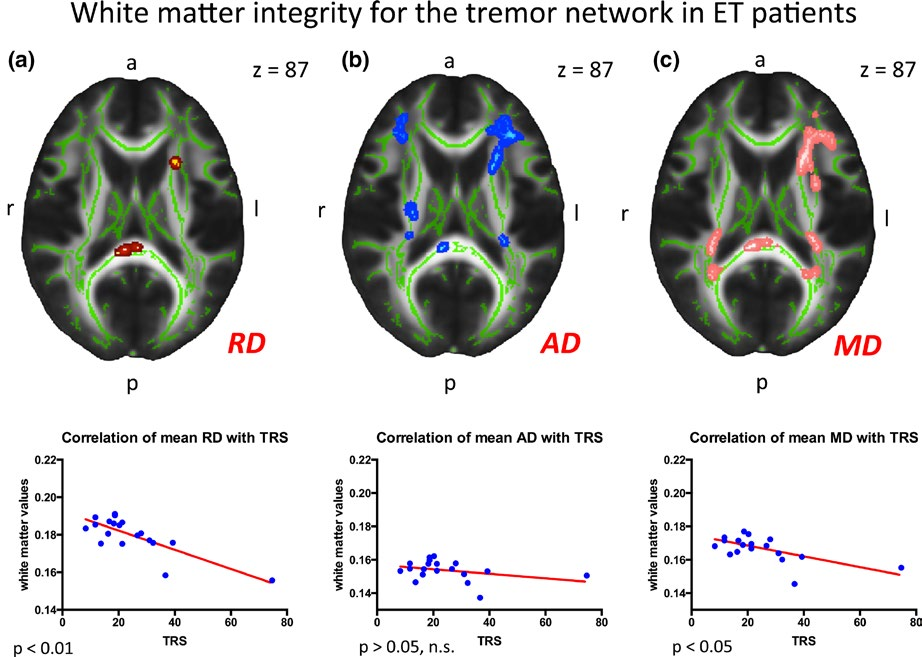
White matter integrity for the tremor network in ET patients. (a–c) Design IIa: Results of the correlation of white matter values with the TRS in axial view (RD‐map [a], AD‐map [b] and MD‐map [c]). Analyses were broadly significant in the cerebral white matter, especially in frontal–parietal regions (*p *< .05). By correlating the mean white matter values with the TRS we found significances in the mean RD [left; *p *< .01] and mean MD [right; *p *< .05], no significance was found for the mean AD [middle; *p *> .05]. RD, radial diffusivity; AD, axonal diffusivity; MD, mean diffusivity

In the ET patients no grey matter involvement regarding the tremor network for corrected statistics was detectable (design IIa). Correlations of white matter values with the TRS (design IIa) were significant in MD‐, RD‐ and AD‐maps (*p *< .05) in: (1) the bilateral corona radiata (specifically anterior parts), (2) the internal capsule, (3) projections to the superior/medial temporal lobe (e.g. superior longitudinal fascicule), (4) the external capsule, and (5) the corpus callosum. These significant correlations were smaller in AD‐ and RD‐ compared to the MD maps (see Figure 2a–c). In contrast, no significant differences were detectable in the FA map.

#### 3.2.3 Grey and white matter differences due to impaired verbal fluency

**Figure 3:**
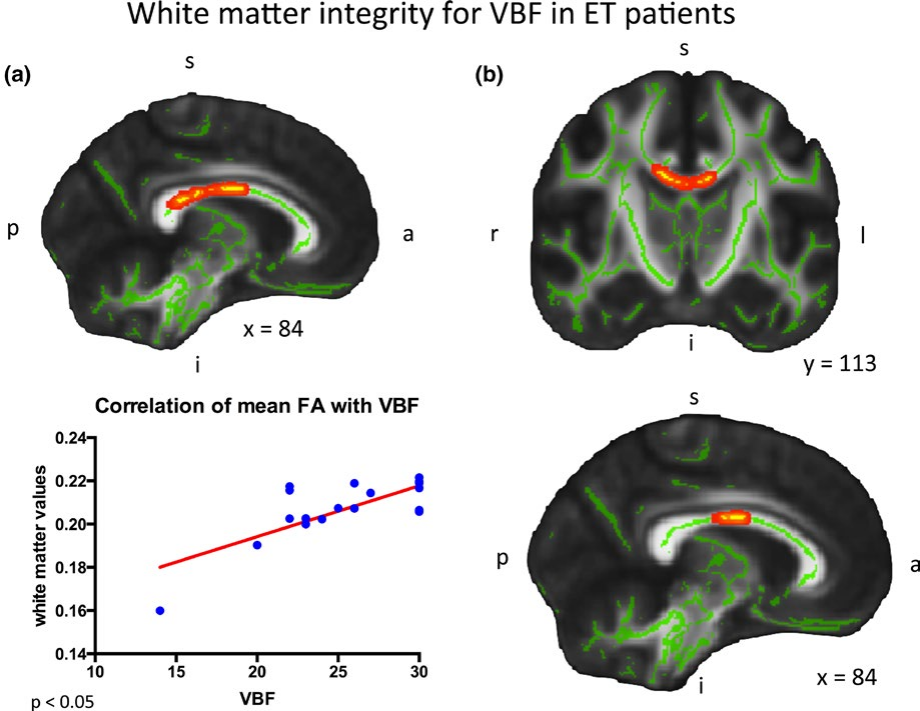
White matter integrity for VBF in ET patients. (a) Design II b: Saggital view (left) and coronal view (right): The correlation with the VBF scores revealed to be highly significant in the corpus callosum regarding the FA map; mean FA values also significantly correlated with decreasing VBF results (*p *< .05). (b) Design II c: Sagittal view of resulting correlation of FA values with VBF with tremor rating scale as covariate of no interest. Only a tendency was found for the latter correlation (*p *< .1). FA, fractional anisotropy; VBF, verbal fluency

In the ET subgroup analysis no grey matter involvement regarding verbal fluency for corrected statistics was detectable (design IIb, design IIc). In the white matter analysis (design IIb) the correlation with VBF values revealed a significant decline in FA values in the corpus callosum in the ET group (*p *< .05; Figure 3a); by additionally including tremor as covariate of no interest (design IIc) the significance in the corpus callosum revealed to be a tendency (*p *< .1; Figure 3b). Both, grey and white matter analysis in the within‐group correlation IIb revealed to be non‐significant in the control group (*p *> .05).

## 4 DISCUSSION

In this study, we report structural differences between ET patients and controls that might be involved in the generation of non‐motor symptoms (Janicki, Cosentino, & Louis, 2013). Three main findings can be summarized as follows: a) ET patients have less grey matter in the predominantly right precuneus compared to healthy controls; in this region greater grey matter volume tended to be associated with greater VBF scores in the patients' group (although non‐significantly) b) in the ET group the corpus callosum shows a significant correlation between white matter and behaviorally‐acquired VBF results; c) ET patients have white matter differences correlating with the increasing tremor score in the MD, RD and AD map, pronounced in fronto‐parietal regions.

Verbal fluency is a common neuropsychological measure of executive functioning and can be subdivided into two different types: (a) phonemic and (b) semantic VBF. Whereas the phonemic VBF means the nomination of words which begin e.g. with “F”, semantic VBF means to take out words of a certain category, as described in the methods section for the DemTect's subtest (“the supermarket”).

**Figure 4:**
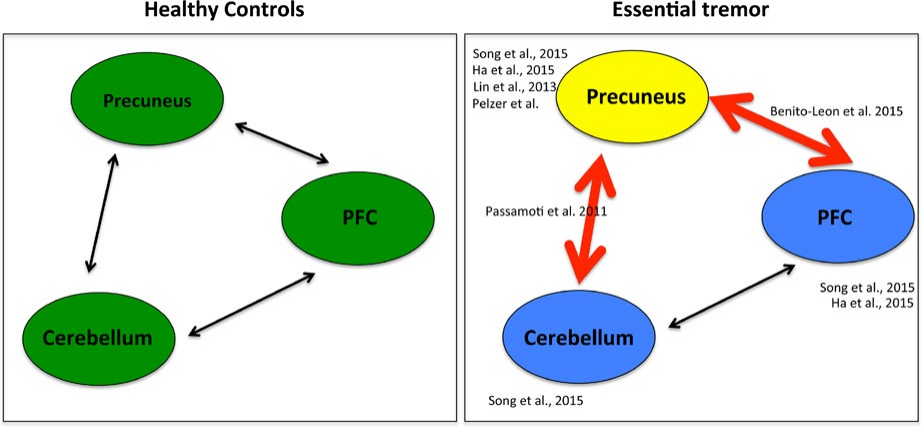
Network changes in ET patients in the non‐motor domain. Compared to healthy controls (left), ET patients (right) show reduced grey matter in the precuneus. Next to these structural differences in the precuneus, the PFC and the cerebellum showed a reduced glucose metabolism and a functionally altered interplay in ET patients. All reporting authors are enlisted next to the specific area/connection they investigated. ET, essential tremor; PFC, prefontal cortex

In healthy controls differences in the performance in semantic VBF tasks have been predicted, for example, by activity changes in the right precuneus (Yin, Zhu, He, Li, & Li, 2015). In ET patients it has been reported, that semantic VBF (next to verbal recall) is specifically affected (Lombardi, Woolston, Roberts, & Gross, 2001; Tröster et al., 2002) and a general relationship between executive functioning (reflected by VBF) and memory (reflected by the verbal recall) exists (Duff, Schoenberg, Scott, & Adams, 2005). These findings are in line with the behavioral results of our study. Until now no structural assignment of behaviorally impaired VBF in ET patients has been performed. In the actual study we connect behavioral findings in ET to structural significances in MRI imaging. We found, that the precuneus seems to play a major role in disturbed semantic VBF in ET patients. Lin et al. (2013) already found a decrease of grey matter in the region of the precuneus in ET patients compared to healthy controls by applying VBM and DARTEL‐VBM analysis before. Also other methodologies already supported an involvement of the precuneus in ET. Two F‐18‐FDG‐PET studies, one by Song, Ha, Yang, and Chung (2015) and one by Ha et al. (2015), confirmed changes in this region with a decrease of glucose utilization in ET patients compared to healthy controls. Next to the precuneus, a decrease in glucose utilization was also found in fronto‐temporo‐occipital lobes and in the study of Song et al. (2015) in the cerebellum. Additionally, functional studies (Benito‐León et al., 2015; Passamonti et al., 2011) found a disturbed interaction of fronto‐parietal regions and the default mode network, of which the precuneus has been described to be part of (Fransson & Marrelec, 2008). In resting state analyses Benito‐León et al. (2015) found increased connectivity in the default mode network and fronto‐parietal network in cognitive processes in ET patients compared to controls and decreased connectivity in the cerebellum and visual networks. Here, network integrity was not only associated with ET severity and ET duration, but also with cognitive ability. In contrast, Passamonti et al. (2011) found a modulation of the resting state networks by the variability in neuropsychological measures: patients with low cognitive scores displayed reduced connectivity between the cerebellum and prefrontal cortex and enhanced connectivity between the cerebellum and the precuneus. They hypothesized that cerebellar neurodegeneration underlying ET is reflected in abnormal communications between key regions responsible for working memory, and that adaptive mechanisms (enhanced response of the cerebellum) occur to limit the expression of cognitive symptoms. The connectivity imbalance between the executive control circuit (including dorsolateral prefrontal cortex, inferior parietal lobule, thalamus) and the default network in patients with ET with low cognitive scores was hypothesized to represent a dysfunction, driven by the cerebellum, in suppressing task‐irrelevant thoughts via focused attention. All of these findings indicate a complex (potentially partial compensatory) interplay in the development of non‐motor symptoms between the prefrontal cortex, cerebellum and the precuneus; the dyshomeostasis in these networks might be caused by the structural impairment of the precuneus, as found in our study (see Figure 4).

In the white matter analysis we found a significant correlation of VBF and the FA values in the corpus callosum (*p *< .05). This is, as far as we know, the first study that describes significant changes in white matter analyses in ET patients with impaired VBF. Due to the fact, that the inclusion of the tremor scores into the correlation (design IIc) has evoked only a tendency (*p *< .1), two possible reasons have to be discussed: (a) the sample is too small to measure structural significances with impaired VBF or (b) impaired VBF is associated with the tremor network although VBF and TRS did behaviorally not significantly correlate with each other (*p *> .05). An involvement of the corpus callosum in the performance of phonemic VBF tasks has however already been described in healthy controls (Hines, Chiu, McAdams, Bentler, & Lipcamon, 1992) and in patients with multiple sclerosis (Pozzilli et al., 1991). Only one recent study exits studying microstructural changes in the white matter of ET patients with cognitive impairment (Bhalsing et al., 2015). These changes correlated with abnormal neuropsychological test scores comprising attention and executive functions, visuo‐spatial functions, working memory and verbal and visual memory in various brain regions, but not semantic VBF. Although all results in the study of Bhalsing et al. (2015) were uncorrected for multiple comparisons, indicating low statistical significance, they already suggested a structural correlate of impaired cognition in ET. Further, especially functional, studies are necessary to reveal the exact role of the affected regions in VBF in ET.

It has been suggested that changes in microstructure are present in patients with ET, e.g. Shin et al. (2008), Klein et al. (2010), Nicoletti et al. (2010). These changes can be detected by an increase or decrease in diffusion parameters, such as the FA. But other markers like the MD, AD, and RD might also reflect impairment in microstructure [for more details see Alexander et al. (2007)]. In our study, we found altered white matter values in the cerebrum in MD, RD, and AD maps that significantly correlated with the TRS. These cerebral findings were comparable to those of Klein et al. (2010). However, we did not find a significant correlation between FA values and the TRS, and no significant differences in the cerebellum compared to controls. According to the literature, the results of TBSS analyses are controversially discussed in ET due to the question of a cerebellar involvement and the localization of changes in the FA map. Although some studies have discussed widespread changes in white matter integrity in the whole brain in ET (Shin et al., 2008), others have reported reduced FA values in the superior cerebellar peduncles and the dentate nucleus (Nicoletti et al., 2010). Klein et al. (2010), however, reported reduced FA in the right‐sided inferior cerebellar peduncles. These differences in the results might be due to the selection of patients. The patients, included in our study, comprised both subtypes, patients with action and intentional tremor; additionally, the disease duration of the individual patients differed. A stronger classification into subgroups might give a solution to these heterogeneous findings of FA and changes in the cerebellum. Accordingly, one recent study analyzed the influence of alterations in LINGO expression, (Leucine‐rich repeat and Immunoglobulindomain‐containing NoGO receptor‐interacting proteins) in ET as a pathological response to ongoing neurodegenerative processes. Compared with controls, LINGO1 protein levels were increased in the cerebellar white matter of Parkinson and ET patients – but for the latter, only when the disease duration exceeded 20 years. In diseased brains, LINGO1 expression seemed to progress along with the disease, being initiated in the cerebellar cortex before reaching the white matter (Delay et al., 2014). Although the mean disease duration was 25.5 years in our study, values were distributed along the Gaussian curve with seven patients with disease duration <20 years, five patients with medium disease duration ≤30 years and seven patients with a long disease duration >30 years. This heterogeneous distribution might be a good explanation for the missing cerebellar white matter involvement in our TBSS analysis in the correlation with the TRS. In addition to the differences in disease duration, possible plasticity effects due to long‐term medication have to be mentioned (Duffau, 2006).

A further subdivision into different subtypes according to disease duration and the occurrence of clinical symptoms is also relevant for grey matter analyses. In accordance with our findings, Quattrone et al. (2008) found no grey matter involvement correlating with the severity of the disease (reflected by the TRS). However, further subdivision of the individual ET‐subtypes revealed a significant correlation with the presence of head tremor and cerebellar atrophy. In contrast, Daniels et al. (2006) reported no grey matter involvement of the cerebellum, although they performed a subgroup analysis of patients with intentional and postural tremor. An automated volumetric method to quantify subcortical atrophy in ET found again a reduction of cerebellar volume in ET (Cerasa et al., 2009); however, no significant linear relationship between clinical variables and adjusted cerebellar volume was found in ET patients with head tremor. In our study, we only included eight patients with predominantly intentional tremor; the others were mixed types, with strong occurrence of the holding tremor component. According to clinical studies there are many variables, indicating the necessity for a stronger subdivision of ET patients in future due to, for example, the age of onset, distribution of tremor, and disease progression (Louis, Ford, & Barnes, 2000). This knowledge should be systematically implemented in future structural imaging studies.

The present results indicate that non‐motor symptoms such as VBF in ET have a structural substrate; their reproduction requires the integration of potential environmental plasticity effects (e.g., medication, age, disease duration), differentiation into individual clinical subtypes and a careful handling with methodological peculiarities of structural MR imaging.

## CONFLICTS OF INTEREST

L.T. received payments as a consultant for Medtronic Inc, Boston Scientific, SAPIENS, St. Jude Medical, Bayer Healthcare, UCB Schwarz Pharma, Archimedes Pharma. L.T. received honoraria as a speaker on symposia sponsored by TEVA Pharma, Lundbeck Pharma, Bracco, Gianni PR, Medas Pharma, UCB Schwarz Pharma, Desitin Pharma, Boehringer Ingelheim, GlaxoSmithKline, Eumecom, Orion Pharma, Medtronic, Boston Scientific, Cephalon, Abott, GE Medical, Archimedes, Bayer, TAD Pharma. C.E. has received speaker's honoraria from Medtronic Inc., Zambon Pharma, TEVA Pharma, UCB Pharma.

## AUTHORS' CONTRIBUTIONS

1. Research project: A. Conception, B. Organization, C. Execution. 2. Statistical Analysis: A. Design, B. Execution, C. Review and Critique. 3. Manuscript Preparation: A. Writing of the first draft, B. Review and Critique. Esther A. Pelzer: 1A, 1B, 2A, 2B, 2C, 3A. Christian Nelles: 1B, 1C, 3B. David J. Pedrosa: 1A, 1B, 1C, 3B. Carsten Eggers: 1C, 3B. Lothar Burghaus: 1C, 3B. Corina Melzer: 2B, 2C, 3B. Marc Tittgemeyer: 1A, 2A, 2C, 3B. Lars Timmermann: 1A, 2A, 2C, 3B.
